# Association between early antenatal care and antenatal care contacts across low-and middle-income countries: effect modification by place of residence

**DOI:** 10.4178/epih.e2021092

**Published:** 2021-11-02

**Authors:** Paschal Awingura Apanga, Maxwell Tii Kumbeni

**Affiliations:** 1 Department of Epidemiology, Biostatistics and Environmental Health, School of Public Health, University of Nevada, Reno, NV, USA; 2 Department of Health Management and Policy, College of Public Health and Human Sciences, Oregon State University, Corvallis, OR, USA

**Keywords:** Early antenatal care, Antenatal contacts, Pregnancy, Residence, Low-and middle-income countries, Effect modification

## Abstract

**OBJECTIVES:**

The relationship between receiving early antenatal care (ANC) and 8 or more antenatal contacts (ANC8+) has not been well characterised across low-and middle-income countries (LMICs). It is also unclear whether the association between early ANC and ANC8+ is modified by a woman’s place of residence. Our primary aim was to assess the relationship between early ANC and ANC8+ and to investigate whether this relationship was modified by place of residence. We also estimated the coverage of ANC8+ across LMICs.

**METHODS:**

We analysed data on 207,388 mothers with a recent live birth using multiple indicator cluster surveys conducted between 2017 and 2020 in 30 LMICS. Modified Poisson regression with robust variance was used to evaluate the relationship between early ANC and ANC8+, whilst adjusting for country, clustering, stratification, and sampling weights. Effect modification by place of residence was assessed on additive and multiplicative scales. A meta-analysis was conducted to pool prevalence estimates of ANC8+ across all countries.

**RESULTS:**

The overall prevalence of ANC8+ was 35.6%, ranging from 1.7% in Madagascar to 99.4% in Belarus. Early ANC was positively associated with ANC8+ (adjusted prevalence ratio [aPR], 2.61; 95% confidence interval [CI], 1.82 to 3.74). There was evidence of positive effect modification on additive (relative excess risk due to interaction, 0.39; 95% CI, 0.35 to 0.44) and multiplicative (aPR, 1.78; 95% CI, 1.08 to 2.95) scales.

**CONCLUSIONS:**

Many LMICs may not have adopted the 2016 World Health Organization guidelines on ANC8+. Receiving early ANC was associated with a higher likelihood of ANC8+, particularly for women in rural areas.

## INTRODUCTION

Antenatal care (ANC) presents a unique and lifesaving window of opportunity for healthcare workers to prevent, detect, and treat pregnancy-related complications during pregnancy [1–3]. In 2016, the World Health Organization (WHO) updated its guidelines on ANC in order to reduce perinatal mortality and improve women’s experience of care [2]. It recommended increasing the number of ANC contacts from a minimum of 4 contacts (ANC4+) to 8 contacts (ANC8+) with emphasis on the timing of initiation of ANC and the quality of services. The current ANC recommendations advocate for the first ANC contact within the first trimester of pregnancy, followed by 2 and 5 additional contacts in the second and third trimesters, respectively [2]. According to the WHO, evidence suggests that more ANC contacts are associated with lower perinatal mortality and higher maternal satisfaction compared to fewer ANC contacts regardless of the resource setting.

ANC coverage is an indicator of access and utilisation of healthcare during pregnancy [4]. It is also key for tracking the global progress towards Sustainable Development Goal (SDG) 3, which aims to reduce the maternal mortality ratio to less than 70 per 100,000 live births and neonatal mortality to as low as 12 per 1,000 live births by 2030 [5]. Despite the significant role of ANC coverage as an indicator for monitoring maternal health globally, relatively little is known about the coverage of ANC8+ across low-and middle-income countries (LMICs). A recent study by Jiwani et al. [6] encompassing 54 LMICs found that the prevalence of ANC8+ coverage was 11.3%, but this proportion included data collected before the 2016 release of the updated WHO guidelines on ANC8+.

Early ANC is defined as initiation of the first ANC contact within the first trimester of pregnancy [7]. It provides healthcare workers the opportunity to discuss the importance of ANC and subsequent ANC appointments with pregnant women. Pregnant women also benefit from health education, an assessment of their gestational age, and identification and treatment of diseases or pregnancy-related complications [8–10]. Early ANC may play an essential role in meeting the WHO 2016 updated guidelines on ANC coverage. However, little is known about the relationship between early ANC and ANC8+ [6,11,12], and previous relevant studies either were not conducted at a global scale or analysed data collected prior to the 2016 release of the updated WHO guidelines on ANC8+. It is also not known whether the relationship between early ANC and ANC8+ is modified by a woman’s place of residence.

Our aim in this study was to characterise the association between early ANC and ANC8+, and to assess whether this relationship is modified by a woman’s place of residence. We hypothesised that women who had early ANC are more likely to have ANC8+ contacts with a healthcare provider, and that this relationship is modified by her place of residence. We also estimated the ANC8+ coverage across 30 LMICs using data from multiple indicator cluster surveys (MICS) collected after the 2016 release of the updated WHO guidelines on ANC.

## MATERIALS AND METHODS

### Study design and study population

We adopted data from MICSs conducted in 30 LMICs between 2017 and 2020. The MICSs are nationally representative household cross-sectional samples, which are country-led with assistance from United Nations Children’s Fund (UNICEF). These surveys usually have a high response rate of 90–95% [13]. The MICSs employ a 2-stage sampling technique in each country, with the first stage involving enumeration areas that are drawn from census files. In the second stage, households are randomly selected from a list of households in each enumeration area. Details of the MICS sampling approach and procedures have been published elsewhere [14,15].

The study population was made up of mothers with a live birth within the last 2 years living in the following 30 LMICs: Algeria, Bangladesh, Belarus, Central African Republic, Costa Rica, Cuba, Democratic Republic of Congo (DRC), Gambia, Ghana, Guinea Bissau, Guyana, Iraq, Kiribati, Kosovo (United Nations Security Council resolution 1244), Kyrgyzstan, Lao People’s Democratic Republic, Lesotho, Madagascar, Mongolia, Montenegro, Nepal, North Macedonia, São Tomé and Príncipe, Serbia, Suriname, Thailand, Togo, Tonga, Tunisia, and Turkmenistan.

### Primary outcome

The primary outcome of interest was mothers who had ANC8+ during pregnancy. Our outcome measure was dichotomised as “1” for mothers who had ANC8+ and “0” for mothers who had fewer than ANC8+ during pregnancy.

### Exposures

The primary exposure was early ANC. Early ANC was defined as a mother having ANC contact with a healthcare provider within the first trimester of pregnancy [7]. Early ANC was dichotomised as mothers who had or did not have early ANC during pregnancy.

### Effect modifier

Place of residence was assessed as an effect modifier. Place of residence was recorded as either rural or urban. Place of residence could plausibly modify the relationship between early ANC and the number of ANC contacts through pathways such as access to healthcare and education. Pregnant women in urban settings are more likely to have higher education and access maternal healthcare services compared to women in rural areas [16,17].

### Covariates

Covariates of interest in our study included: age, marital status, mothers’ educational level, household wealth, planned pregnancy status, parity, and perceived domestic violence. Covariates were categorised as follows: marital status (married/cohabitation, single); mothers’ educational level (≥secondary education, <secondary education); place of residence (rural, urban); and parity (multiparous, primiparous). Other covariates included age, which was assessed as a continuous variable, and household wealth, which was categorized into wealth quintiles (poorest, poor, middle, rich, and richest). Household wealth quintiles are a composite indicator of wealth derived from principal component analysis using household assets [14]. Perceived domestic violence was considered present if a mother reported that her husband/partner unjustifiably beat/hit her if she performed any of the following actions: if she went out without telling him; if she neglected the children; if she argued with him; if she refused to have sex with him, and if she burnt the food.

### Data analysis

Descriptive statistics were used to assess the study population. Whilst categorical variables were presented as counts and percentages, continuous variables were presented as mean and standard deviation.

Descriptive statistics were also used to assess the coverage (i.e., prevalence) of ANC8+ and mothers who had ANC4+ by country. We conducted a random-effects meta-analysis with inverse variance weighting to pool estimates of our results across all countries. Heterogeneity across countries was reported using the I^2^ statistic. An I^2^ >50% was deemed to indicate substantial heterogeneity, whilst an I^2^ >75% indicated considerable heterogeneity [18]. We presented results by each country and overall using forest plots.

To assess the association between early ANC and ANC8+ and whether this relationship was modified by place of residence, we conducted a series of regression models using modified Poisson regression with robust error variance. Consistent with MICS guidelines and to ensure the representativeness of our data [19], each of the models was adjusted for the sampling design, taking into account the country, sampling weight, clustering, and stratification variables.

To assess the relationship between early ANC and ANC8+, we conducted univariate analyses for all variables (i.e., exposures and covariates) to assess the relationship between each variable and ANC8+. Variables with p-values of less than 0.2 were included in the multivariable model to assess the association between early ANC and ANC8+.

To assess effect modification by place of residence, we used a model similar to the multivariable model used to assess the relationship between early ANC and ANC8+, but included an interaction term between early ANC and place of residence (early ANC* place of residence). Effect modification was assessed on additive and multiplicative scales.

We used relative excess risk due to interaction (RERI) to assess effect modification on an additive scale, as this is the most appropriate public health measure for assessing effect modification on an additive scale [20,21]. The RERI and corresponding 95% confidence intervals (CIs) were estimated using the “MOVER” approach proposed by Zou [22]. An estimate greater than 0 signified positive effect modification, whilst an estimate less than 0 signified negative effect modification [23]. With regards to effect modification on the multiplicative scale, an estimate greater than 1 was an indication of positive effect modification, whilst an estimate less than 1 was considered negative effect modification [23]. We presented our effect modification results in the format recommended by Knol & VanderWeele [24].

We also conducted a sensitivity analysis to determine whether our findings on effect modification were sensitive to a different categorization of our outcome. Therefore, we assessed whether the relationship between early ANC and ANC4+ (i.e., the previous ANC recommendation) was modified by place of residence.

The variables for our analyses were selected based on factors reported in previous studies [11,12,25] and availability in the MICS datasets. Missing data on individuals were excluded, as this was fewer than 2% of participants. We used Stata/SE version 16 (StataCorp., College Station, TX, USA) to conduct the meta-analysis, whilst SAS version 9.4 (SAS Institute Inc., Cary, NC, USA) was used for the descriptive statistics and regression analyses.

### Ethics statement

This study did not require ethical approval as we used de-identified secondary data that are publicly available. Details of the ethical approval for the MICS datasets have been published elsewhere [14].

## RESULTS

The study population was made up of 207,388 mothers with a live birth in the last 2 years (Table 1). The flow diagram for the screening and selection of the 30 MICS datasets from LMICs that were included in this study is shown in Supplementary Material 1. A country was considered an LMIC based on the World Bank’s classification [26]. We screened a total of 343 surveys conducted between January 1993 to December 2020 for our study. This study excluded surveys that were conducted before the release of the 2016 revised WHO guidelines on ANC8+ [2]. We also excluded countries that did not have available data or countries with data limited only to select counties/regions (Supplementary Material 1). Therefore, we identified 30 surveys conducted between January 2017 and December 2020 for inclusion in our study.

The mean age of mothers in our study was 28 years (Table 1), and this ranged from 26 years to 32 years across each of the countries (Supplementary Material 2). Many of the mothers (27.5%) in our study were in the poorest wealth quintile (Table 1). Overall, most mothers (61.9%) resided in urban areas (Table 1), although 15 of the countries had a majority of the mothers living in rural areas (Supplementary Material 2). The prevalence of early ANC ranged from 16.9% in DRC to 97.0% in Serbia (Supplementary Material 2).

**Figure 1:**
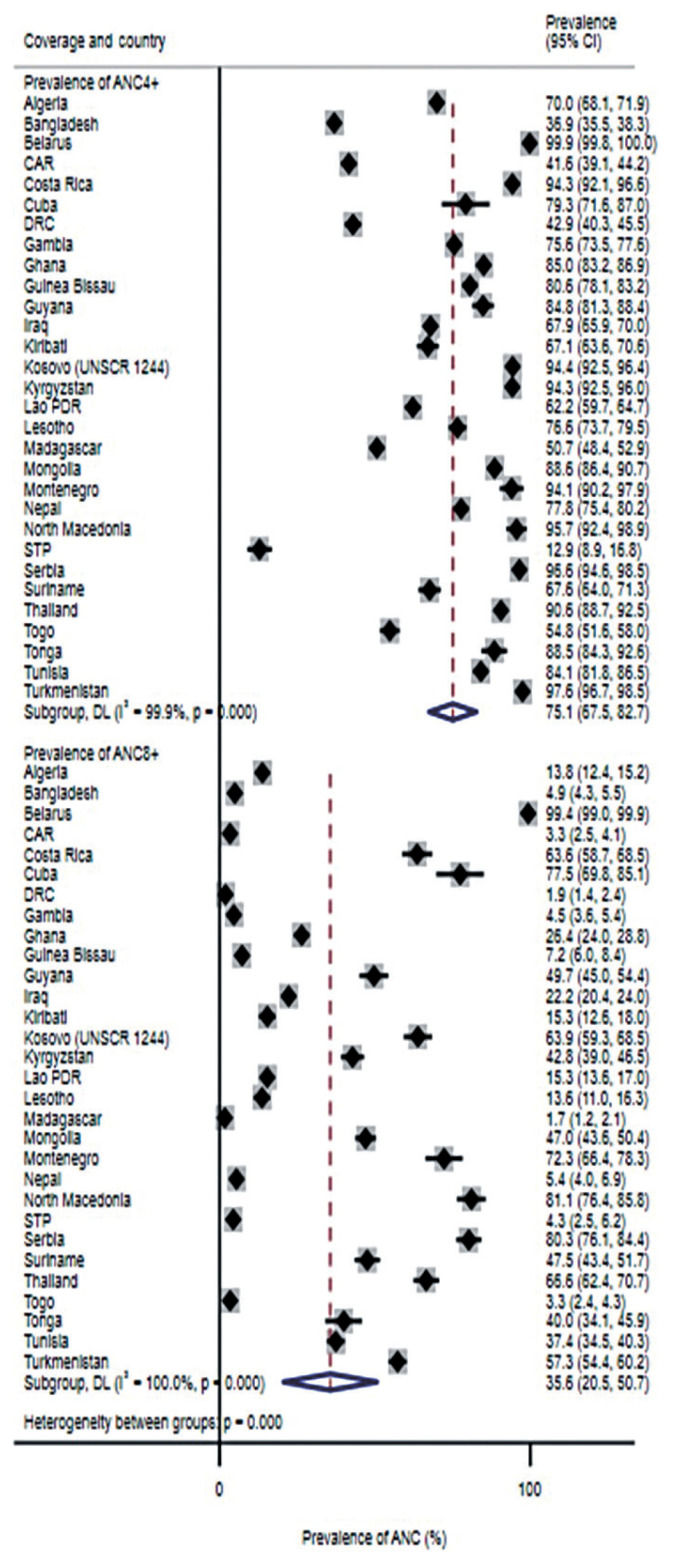
Coverage of 4 or more antenatal care contacts (ANC4+) and 8 or more antenatal care contacts (ANC8+) by country and overall. CI, confidence interval; CAR, Central African Republic; DRC, Democratic Republic of the Congo; Lao PDR, Lao People’s Democratic Republic; STP, São Tomé and Príncipe.

Our meta-analysis results showed that the overall prevalence of ANC8+ was 35.6% (95% CI, 20.5 to 50.7) with substantial heterogeneity across all countries (I^2^=100%, p<0.001) (Figure 1). The coverage of ANC8+ ranged from 1.7% in Madagascar to 99.4% in Belarus, and ANC8+ prevalence was at least 50% in 9 of the 30 countries. The overall prevalence of ANC4+ was 75.1% (95% CI, 67.5 to 82.7) with substantial heterogeneity across all countries (I^2^=99.9%; p<0.001) (Figure 1) (Supplementary Material 3).

The prevalence of ANC8+ among mothers who had early ANC was 2.61 times the prevalence of ANC8+ among mothers who did not receive early ANC (adjusted prevalence ratio [aPR], 2.61; 95% CI, 1.82 to 3.74) (Table 2). We also found that mothers in rich households had a 26% higher prevalence of ANC8+ than mothers in the poorest households (aPR, 1.26; 95% CI, 1.06 to 1.51). The prevalence of ANC8+ was 24% higher among mothers whose pregnancies were planned compared to those who had unplanned pregnancies (aPR, 1.24; 95% CI, 1.06 to 1.46) (Table 2).

The results for effect modification showed strong evidence of a positive effect modification by place of residence on the relationship between early ANC and ANC8+ on the multiplicative (aPR, 1.78; 95% CI, 1.08 to 2.95), and additive (RERI, 0.39; 95% CI, 0.35 to 0.44), scales (Table 3). Among mothers who did not have early ANC, the prevalence of ANC8+ was 45% lower among those who resided in rural areas than among those who resided in urban areas (aPR, 0.55; 95% CI, 0.33 to 0.90). However, among mothers residing in rural areas, the prevalence of ANC8+ among those who had early ANC was 4.02 times the prevalence of ANC8+ among mothers who did not have early ANC (aPR, 4.02; 95% CI, 2.93 to 5.52) (Table 3).

Our sensitivity analysis on whether the association between early ANC and ANC4+ was modified by place of residence also showed similar results, with positive effect modification on both multiplicative and additive scales (Supplementary Material 4).

## DISCUSSION

The primary aim of this study was to assess the relationship between early ANC and ANC8+ and to investigate whether this relationship was modified by a woman’s place of residence. We also estimated the coverage of ANC8+ among 207,388 mothers with a live birth across 30 LMICs. The prevalence of ANC8+ was low in many of the countries in our study. Our analysis also showed that mothers who received early ANC were more likely to attain ANC8+, and this relationship was modified by the place of residence on multiplicative and additive scales.

We observed that only 9 of the 30 countries reported at least 50% coverage of ANC8+, with an overall prevalence of 36.5%. The overall prevalence of ANC8+ in our study was higher than that reported by Jiwani et al. [6] across 54 LMICs. The difference in our findings was probably because the study by Jiwani et al. [6] included data collected before the 2016 release of the updated WHO guidelines on ANC. This could have led to an underestimation of the prevalence of ANC8+, as it was not recommended before 2016. The prevalence of ANC8+ in our study was also higher than the overall prevalence of ANC8+ across 15 LMICs reported in a recent study [27]. The low compliance with ANC8+ across many of the countries in our study could have been due to several reasons. Some countries might not have adopted the updated WHO guidelines on ANC8+ [11,12,28]. Policy adoption and implementation involve administrative and logistical adjustments, including training of healthcare providers, which may require more time to implement in some countries. Pregnant women may also be unaware of recent guidelines on ANC8+. Our findings on the coverage of ANC8+ provide insight on LMICs that might need support to increase the coverage of ANC8+.

Our study also found that among all mothers, early ANC was associated with ANC8+. Our finding aligns well with previous studies in Benin and Nigeria [11,12]. Among mothers who did not receive early ANC, those residing in the rural areas were less likely to attain ANC8+ than those in urban areas. However, early ANC among mothers in rural areas was associated with a higher prevalence of ANC8+. Our findings suggest that to fulfil the updated WHO guidelines on ANC, pregnant women should be encouraged to receive early ANC, particularly women in rural areas. Therefore, public health programmes/interventions targeted at increasing the coverage of ANC8+ may need to prioritize pregnant women in rural communities to receive early ANC.

Our finding on the association between mothers in rich households and ANC8+ is consistent with previous research [28]. Not surprisingly, mothers who had planned their pregnancies were more likely to have ANC8+ compared to those with unplanned pregnancies. This finding has also been reported previously, but that study analysed data before the 2016 release of the updated WHO guidelines [29].

Our study has several strengths and limitations that should be acknowledged. The use of MICS data, which is nationally representative, enables our findings to be generalizable to the countries in our study and potentially to other LMICs. A major limitation is that because of the cross-sectional design of our study, our findings cannot be interpreted causally. Our outcome measure was self-reported and may be subject to recall bias, but we have no reason to expect recall to be different between mothers who had early ANC and mothers who did not. Another limitation is that we controlled for a limited number of confounding factors, and therefore our findings may still be subject to residual confounding (e.g., data on high-risk pregnancies was not available in the MICS datasets).

In conclusion, this is the first study to assess the relationship between early ANC and ANC8+ across LMICs using only data collected after the 2016 release of the updated WHO guidelines on ANC. It is also the first to provide evidence of effect modification by a woman’s place of residence on the association between early ANC and ANC8+. We recommend the promotion of early ANC among all women, particularly those residing in rural areas, as this may help achieve the recommendations of the updated WHO guidelines for a positive pregnancy experience. Educating men on the importance of early ANC may also be essential for increasing early ANC coverage. We also recommend providing incentives to pregnant women who receive early ANC, as this may play an important role in increasing the coverage of early ANC.

## Figures and Tables

**Table 1:** Characteristics of the study population across 30 low-and middle-income countries (n=207,388)

Characteristics	n (%) or n
Age, mean±standard deviation (yr)	28.0±10.1

Marital status	
Single	49,976 (24.1)
Married/cohabitation	157,395 (75.9)
Missing	17

Education	
<Secondary	60,801 (29.3)
≥Secondary	146,576 (70.7)
Missing	11

Household wealth (quintile)	
Poorest	56,938 (27.5)
Poor	44,357 (21.4)
Middle	39,072 (18.8)
Rich	36,088 (17.4)
Richest	30,933 (14.9)

Place of residence	
Urban	128,400 (61.9)
Rural	78,988 (38.1)

Planned pregnancy	
Unplanned	80,852 (39.1)
Planned	125,967 (60.9)
Missing	569

Parity	
Primiparous	79,081 (38.1)
Multiparous	128,306 (61.9)
Missing	1

Early ANC	
No	52,603 (25.4)
Yes	154,136 (74.6)
Missing	650

Perceived domestic violence	
No	173,177 (84.8)
Yes	31,037 (15.2)
Missing	3,174

Values are presented as number (%) or number.

ANC, antenatal care.

**Table 2:** Association between early ANC and coverage of ANC8+

Variables	Univariate models	p-value^1^	Multivariable model	p-value^1^
Early ANC				
No	1.00 (reference)		1.00 (reference)	
Yes	2.73 (1.92, 3.89)	<0.01	2.61 (1.82, 3.74)	<0.01

Age	1.01 (1.00, 1.02)	0.04	1.00 (0.99, 1.02)	0.50

Marital status				
Single	1.00 (reference)		1.00 (reference)	
Married/cohabitation	1.22 (1.03, 1.44)	0.02	1.04 (0.88, 1.22)	0.67

Education				
<Secondary	1.00 (reference)		1.00 (reference)	
≥Secondary	1.27 (1.07, 1.50)	0.01	1.13 (0.95, 1.35)	0.18

Household wealth (quintile)				
Poorest	1.00 (reference)		1.00 (reference)	
Poor	1.25 (1.05, 1.50)	0.01	1.18 (1.00, 1.39)	0.05
Middle	1.33 (1.12, 1.58)	<0.01	1.17 (1.00, 1.37)	0.06
Rich	1.48 (1.24, 1.78)	<0.01	1.26 (1.06, 1.51)	0.01
Richest	1.37 (1.03, 1.84)	0.03	1.11 (0.82, 1.52)	0.50

Place of residence				
Urban	1.00 (reference)		1.00 (reference)	
Rural	0.92 (0.81, 1.04)	0.19	0.94 (0.84, 1.06)	0.33

Planned pregnancy				
Unplanned	1.00 (reference)		1.00 (reference)	
Planned	1.33 (1.13, 1.56)	<0.01	1.24 (1.06, 1.46)	0.01

Parity				
Primiparous	1.00 (reference)		-	-
Multiparous	1.02 (0.88, 1.18)	0.78	-	

Perceived domestic violence				
No	1.00 (reference)		1.00 (reference)	
Yes	0.83 (0.66, 1.06)	0.13	0.89 (0.70, 1.13)	0.35

Values are presented as prevalence ratio (95% confidence interval).

ANC, antenatal care; ANC8+, 8 or more antenatal care contacts.

1 p-value <0.05 indicates statistical significance.

**Table 3:** Effect modification^1^ of the association between early ANC and ANC8+ by place of residence^2^

Place	No early ANC		Early ANC		PR (95% CI) comparing ANC8+ coverage with early ANC vs. not within strata of place of residence
	
	With/Without outcome (n)	PR (95% CI)	With/Without outcome (n)	PR (95% CI)	
Urban	762/10,199	1.00 (reference)	8,441/11,866	2.25 (1.47, 3.44)	2.25 (1.47, 3.44)
p-value				<0.01	<0.01

Rural	898/25,666	0.55 (0.33, 0.90)	6,832/15,073	2.19 (1.42, 3.38)	4.02 (2.93, 5.52)
p-value		0.02		<0.01	<0.01

ANC, antenatal care; ANC8+, 8 or more antenatal care contacts; PR, prevalence ratio; CI, confidence interval; RERI, relative excess risk due to interaction.

1 Measure of effect modification on additive scale: RERI (95% CI) = 0.39 (0.35, 0.44); p<0.01; Measure of effect modification on multiplicative scale: ratio of PRs (95% CI) = 1.78 (1.08, 2.95); p=0.02.

2 PRs are adjusted for age, marital status, education, household wealth, planned pregnancy and perceived domestic violence.
