# Supplementary material for: Association between early antenatal care and antenatal care contacts across low-and middle-income countries: effect modification by place of residence
Source: Epidemiol Health. 2021 Nov 2;43:e2021092. doi: 10.4178/epih.e2021092 (PMC8920740; doi:10.4178/epih.e2021092)
Supplement: Supplementary file 1 [file epih-43-e2021092-suppl1.docx]

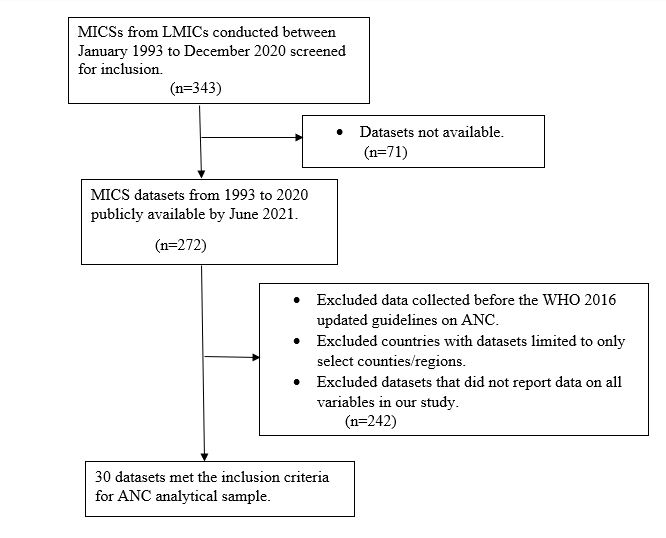


Supplementary Material 1. Flow diagram of nationally representative household surveys screened for study inclusion and analysis.
