# Supplementary material for: Association between early antenatal care and antenatal care contacts across low-and middle-income countries: effect modification by place of residence
Source: Epidemiol Health. 2021 Nov 2;43:e2021092. doi: 10.4178/epih.e2021092 (PMC8920740; doi:10.4178/epih.e2021092)
Supplement: Supplementary file 3 [file epih-43-e2021092-suppl3.docx]

Supplementary Material 3. Coverage of antenatal care by country

| Country | ANC4+ (95% CI) | ANC8+ (95% CI) |
| --- | --- | --- |
| Algeria | 70% (68.1%, 71.9%) | 13.8% (12.4%, 15.2%) |
| Bangladesh | 36.9% (35.5%, 38.3%) | 4.9% (4.3%, 5.5%) |
| Belarus | 99.9% (99.8%, 100%) | 99.4% (99%, 99.9%) |
| CAR | 41.6% (39.1%, 44.2%) | 3.3% (2.5%, 4.1%) |
| Costa Rica | 94.3% (92.1%, 96.6%) | 63.6% (58.7%, 68.5%) |
| Cuba | 79.3% (71.6%, 87%) | 77.5% (69.8%, 85.1%) |
| DRC | 42.9% (40.3%, 45.5%) | 1.9% (1.4%, 2.4%) |
| Gambia | 75.6% (73.5%, 77.6%) | 4.5% (3.6%, 5.4%) |
| Ghana | 85% (83.2%, 86.9%) | 26.4% (24%, 28.8%) |
| Guinea Bissau | 80.6% (78.1%, 83.2%) | 7.2% (6%, 8.4%) |
| Guyana | 84.8% (81.3%, 88.4%) | 49.7% (45%, 54.4%) |
| Iraq | 67.9% (65.9%, 70%) | 22.2% (20.4%, 24%) |
| Kiribati | 67.1% (63.6%, 70.6%) | 15.3% (12.6%, 18%) |
| Kosovo (UNSCR 1244) | 94.4% (92.5%, 96.4%) | 63.9% (59.3%, 68.5%) |
| Kyrgyzstan | 94.3% (92.5%, 96%) | 42.8% (39%, 46.5%) |
| Lao PDR | 62.2% (59.7%, 64.7%) | 15.3% (13.6%, 17%) |
| Lesotho | 76.6% (73.7%, 79.5%) | 13.6% (11%, 16.3%) |
| North Macedonia | 95.7% (92.4%, 98.9%) | 81.1% (76.4%, 85.8%) |
| Madagascar | 50.7% (48.4%, 52.9%) | 1.7% (1.2%, 2.1%) |
| Mongolia | 88.6% (86.4%, 90.7%) | 47% (43.6%, 50.4%) |
| Montenegro | 94.1% (90.2%, 97.9%) | 72.3% (66.4%, 78.3%) |
| Nepal | 77.8% (75.4%, 80.2%) | 5.4% (4%, 6.9%) |
| STP | 12.9% (8.9%, 16.8%) | 4.3% (2.5%, 6.2%) |
| Serbia | 96.6% (94.6%, 98.5%) | 80.3% (76.1%, 84.4%) |
| Suriname | 67.6% (64%, 71.3%) | 47.5% (43.4%, 51.7%) |
| Thailand | 90.6% (88.7%, 92.5%) | 66.6% (62.4%, 70.7%) |
| Togo | 54.8% (51.6%, 58%) | 3.3% (2.4%, 4.3%) |
| Tonga | 88.5% (84.3%, 92.6%) | 40% (34.1%, 45.9%) |
| Tunisia | 84.1% (81.8%, 86.5%) | 37.4% (34.5%, 40.3%) |
| Turkmenistan | 97.6% (96.7%, 98.5%) | 57.3% (54.4%, 60.2%) |

CAR: Central African Republic; DRC: Democratic Republic of the Congo;

Lao PDR: Lao People's Democratic Republic; STP: São Tomé and Príncipe

ANC8+: eight or more antenatal care contacts

ANC4+: four or more antenatal care contacts
