# Supplementary material for: Association between early antenatal care and antenatal care contacts across low-and middle-income countries: effect modification by place of residence
Source: Epidemiol Health. 2021 Nov 2;43:e2021092. doi: 10.4178/epih.e2021092 (PMC8920740; doi:10.4178/epih.e2021092)
Supplement: Supplementary file 4 [file epih-43-e2021092-suppl4.docx]

Supplementary Material 4. Effect modification of the association between early ANC and ANC4+ by place of residence

|  | No early ANC | |  | Early ANC | |  |
| --- | --- | --- | --- | --- | --- | --- |
|  | N with/without outcome | PR (95% CI) |  | N with/without outcome | PR (95% CI) | PR (95% CI) comparing ANC4+ coverage with early ANC vs. not within strata of place of residence |
| Urban | 9698/16866 | 1.00 (Reference) |  | 18139/3766 | 1.39 (1.21,1.60)  *p<0.01* | 1.39 (1.21,1.60)  *p<0.01* |
| Rural | 5628/5333 | 0.80 (0.70,0.90)  *p<0.01* |  | 17638/2669 | 1.36 (1.19,1.56)  *p<0.01* | 1.71 (1.59,1.85)  *p<0.01* |

Measure of effect modification on additive scale: RERI (95% CI) = 0.18 (0.15, 0.20); *p<0.01*

Measure of effect modification on multiplicative scale: ratio of PRs (95% CI) = 1.23 (1.08, 1.40); p<0.01

Prevalence ratios (PRs) are adjusted for age, marital status, education, household wealth, planned pregnancy and perceived domestic violence

ANC: antenatal care; ANC4+: four or more antenatal care contacts
